# 2-[2-(2-Nitro­phen­yl)-4,5-diphenyl-1*H*-imidazol-1-yl]-3-phenyl­propan-1-ol

**DOI:** 10.1107/S1600536814008770

**Published:** 2014-04-30

**Authors:** Yizhen Li, Pu Mao, Yongmei Xiao, Liangru Yang, Lingbo Qu

**Affiliations:** a School of Chemistry and Chemical engineering, Henan University of Technology, Zhengzhou 450001, People’s Republic of China

## Abstract

In the title compound, C_30_H_25_N_3_O_3_, the central imidazole ring forms dihedral angles of 77.34 (6), 12.56 (6) and 87.04 (6)°, respectively, with the *o*-nitro­benzene ring and the phenyl substituents in the 5- and 4-positions. The mol­ecular conformation is stabilized by weak intra­molecular C—H⋯π inter­actions. In the crystal, mol­ecules are linked by O—H⋯N hydrogen bonds, forming chains running parallel to the *b*-axis direction.

## Related literature   

For the synthesis of imidazole derivatives, see: Ding *et al.* (2005 ▶); Heightman & Vasella (1999 ▶); Wasserscheid & Keim (2000 ▶). For related compounds synthesized by our group, see: Gao, Yang *et al.* (2013 ▶); Gao, Wang *et al.* (2013 ▶); Mao *et al.* (2010 ▶); Yang *et al.* (2012 ▶); Xiao *et al.* (2012 ▶).
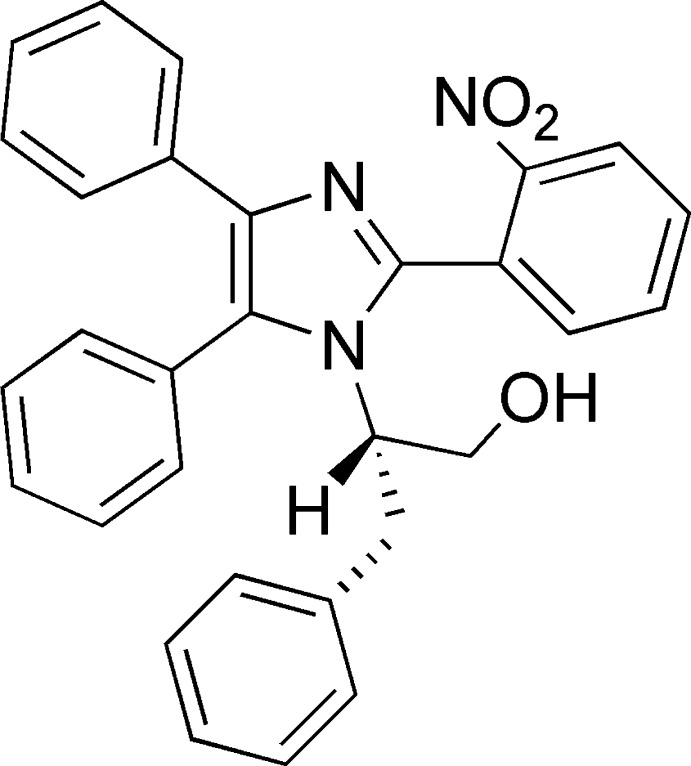


## Experimental   

### 

#### Crystal data  

C_30_H_25_N_3_O_3_
*M* *_r_* = 475.53
Orthorhombic, 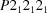

*a* = 10.54812 (16) Å
*b* = 12.77836 (19) Å
*c* = 18.4800 (3) Å
*V* = 2490.87 (6) Å^3^
*Z* = 4
Cu *K*α radiation
μ = 0.67 mm^−1^
*T* = 291 K
0.25 × 0.22 × 0.20 mm

#### Data collection  

Agilent Xcalibur (Eos, Gemini) diffractometer
Absorption correction: multi-scan (*CrysAlis PRO*; Agilent, 2011 ▶) *T*_min_ = 0.638, *T*_max_ = 1.000
23649 measured reflections
4460 independent reflections
4263 reflections with *I* > 2σ(*I*)
*R*_int_ = 0.054

#### Refinement  

*R*[*F* ^2^ > 2σ(*F* ^2^)] = 0.038
*wR*(*F* ^2^) = 0.111
*S* = 1.05
4460 reflections
330 parameters
H atoms treated by a mixture of independent and constrained refinement
Δρ_max_ = 0.16 e Å^−3^
Δρ_min_ = −0.14 e Å^−3^
Absolute structure: Flack (1983 ▶); 1925 Friedel pairs
Absolute structure parameter: 0.2 (2)

### 

Data collection: *CrysAlis PRO* (Agilent, 2011 ▶); cell refinement: *CrysAlis PRO*; data reduction: *CrysAlis PRO*; program(s) used to solve structure: *SHELXS97* (Sheldrick, 2008 ▶); program(s) used to refine structure: *SHELXL97* (Sheldrick, 2008 ▶); molecular graphics: *OLEX2* (Dolomanov *et al.*, 2009 ▶); software used to prepare material for publication: *OLEX2*.

## Supplementary Material

Crystal structure: contains datablock(s) I, global. DOI: 10.1107/S1600536814008770/rz5116sup1.cif

Structure factors: contains datablock(s) I. DOI: 10.1107/S1600536814008770/rz5116Isup2.hkl

Supporting information file. DOI: 10.1107/S1600536814008770/rz5116Isup3.cml

CCDC reference: 997919

Additional supporting information:  crystallographic information; 3D view; checkCIF report

## Figures and Tables

**Table 1:** Hydrogen-bond geometry (Å, °) *Cg*1 and *Cg*2 are the centroids of the C9–C14 and C22–C27 rings, respectively.

*D*—H⋯*A*	*D*—H	H⋯*A*	*D*⋯*A*	*D*—H⋯*A*
C5—H5⋯*Cg*1	0.93	2.84	3.715 (2)	157
C21—H21⋯*Cg*2	0.93	2.81	3.501 (2)	132
O3—H3⋯N1^i^	0.90 (3)	1.89 (3)	2.7935 (17)	179 (3)

Symmetry code: (i) 
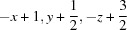
.

**Fig. 1.**
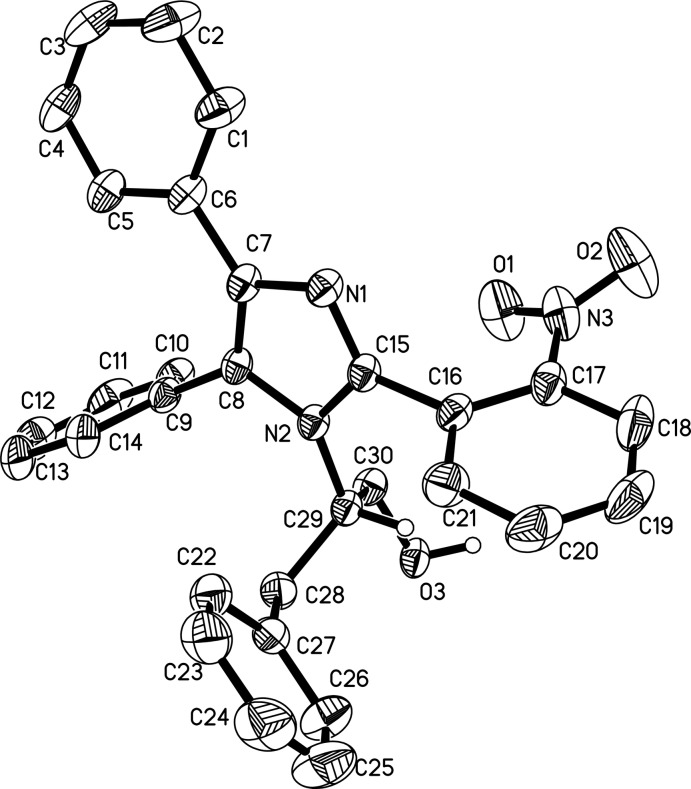
The molecular structure of the title compound showing 30% probability displacement ellipsoids. Hydrogen atoms, but those associated to the chiral C29 carbon atom and hydroxyl group, are omitted for clarity.

## supplementary crystallographic information

### 1. Comment

Imidazole and its derivatives attracted research interest due to their important roles in the field of biology, medicine and chemistry. Imidazoles containing chiral *N*-substituent have high potentiality for application in coordination chemistry and transition metal catalysis (Ding *et al.*, 2005; Heightman & Vasella, 1999; Wasserscheid & Keim, 2000). Our group is interested in the synthesis and application of chiral imidazolium compounds derived from natural amino acids (Gao, Yang *et al.*, 2013; Gao, Wang *et al.*, 2013; Mao *et al.*, 2010; Yang *et al.*, 2012; Xiao *et al.*, 2012). Here we present the synthesis of a chiral nirtrophenyl-substituted imidazole derivative obtained from the condensation of a chiral aminoalcohol, nitrobenzaldehyde, ammonium acetate and benzyl. The synthetic procedure provides valuable information for the research and development of novel chiral catalysts.

The molecular structure of the title compound is shown in Figure 1. As expected, the imidazole core (C7/C8/N2/C15/N1) is essentially planar (r.m.s. deviation = 0.0056 Å). The dihedral angles it forms with the *o*-nitrobenzene ring and the two phenyl substituents (C1—C6, C9—C14) are 77.34 (6), 12.56 (6) and 87.04 (6)°, respectively. Two intramolecular C—H···π interactions stabilizing the molecular conformation are observed (Table 1). In the crystal, molecules are linked by O—H···N hydrogen bonds (Table 1), forming chains running parallel to the *b* axis.

### 2. Experimental

*L*-Phenylalaninol (15.1 g, 0.1 mol) was added to the solvent (CH_3_OH, 200 mL) with ammonium acetate (7.7 g, 0.1 mol) and dibenzoyl (21.0 g, 0.1 mol) in a three-neck flask. The system was stirred until *L*-phenylalaninol was dissolved completely, affording a transparent dark yellow solution. The flask was then put into an ice bath and *o*-nitrobenzaldehyde (15.1 g, 0.1 mol) in MeOH (20 ml) was added dropwise to the solution. The mixture was heated to 65°C for 12 h. The solvent was eliminated by vacuum rotary evaporation and the crude product obtained purified using column chromatography (ethyl acetate/ethanol/triethylamine, 10:1:0.1 *v*/*v*/*v*). Crystallization of the product by slow evaporation of a methanol/diethyl ether solution (1:1 *v*/*v*) afforded crystals of the title compound suitable for X-ray analysis.

### 3. Refinement

The hydroxyl H atom was located in a difference Fourier map and refined freely. All other H atoms were placed in calculated positions with C—H = 0.93–0.98 Å and refined as riding, with *U*_iso_(H) = 1.2*U*_eq_(C).

### Crystal data

**Table:**

C_30_H_25_N_3_O_3_	*D*_x_ = 1.268 Mg m^−^^3^
*M**_r_* = 475.53	Cu *K*α radiation, λ = 1.5418 Å
Orthorhombic, *P*2_1_2_1_2_1_	Cell parameters from 12200 reflections
*a* = 10.54812 (16) Å	θ = 4.2–72.5°
*b* = 12.77836 (19) Å	µ = 0.67 mm^−^^1^
*c* = 18.4800 (3) Å	*T* = 291 K
*V* = 2490.87 (6) Å^3^	, yellow
*Z* = 4	0.25 × 0.22 × 0.20 mm
*F*(000) = 1000	

### Data collection

**Table:**

Agilent Xcalibur (Eos, Gemini) diffractometer	4460 independent reflections
Radiation source: Enhance (Cu) X-ray Source	4263 reflections with *I* > 2σ(*I*)
Graphite monochromator	*R*_int_ = 0.054
Detector resolution: 16.2312 pixels mm^-1^	θ_max_ = 67.1°, θ_min_ = 4.2°
ω scans	*h* = −12→12
Absorption correction: multi-scan (*CrysAlis PRO*; Agilent, 2011)	*k* = −15→14
*T*_min_ = 0.638, *T*_max_ = 1.000	*l* = −22→21
23649 measured reflections	

### Refinement

**Table:**

Refinement on *F*^2^	Hydrogen site location: inferred from neighbouring sites
Least-squares matrix: full	H atoms treated by a mixture of independent and constrained refinement
*R*[*F*^2^ > 2σ(*F*^2^)] = 0.038	*w* = 1/[σ^2^(*F*_o_^2^) + (0.0732*P*)^2^ + 0.1165*P*] where *P* = (*F*_o_^2^ + 2*F*_c_^2^)/3
*wR*(*F*^2^) = 0.111	(Δ/σ)_max_ = 0.001
*S* = 1.05	Δρ_max_ = 0.16 e Å^−^^3^
4460 reflections	Δρ_min_ = −0.14 e Å^−^^3^
330 parameters	Extinction correction: *SHELXL97* (Sheldrick, 2008), Fc^*^=kFc[1+0.001xFc^2^λ^3^/sin(2θ)]^-1/4^
0 restraints	Extinction coefficient: 0.0020 (3)
Primary atom site location: structure-invariant direct methods	Absolute structure: Flack (1983); 1925 Friedel pairs
Secondary atom site location: difference Fourier map	Absolute structure parameter: 0.2 (2)

### Special details

**Table:**

Geometry. All e.s.d.'s (except the e.s.d. in the dihedral angle between two l.s. planes) are estimated using the full covariance matrix. The cell e.s.d.'s are taken into account individually in the estimation of e.s.d.'s in distances, angles and torsion angles; correlations between e.s.d.'s in cell parameters are only used when they are defined by crystal symmetry. An approximate (isotropic) treatment of cell e.s.d.'s is used for estimating e.s.d.'s involving l.s. planes.
Refinement. Refinement of *F*^2^ against ALL reflections. The weighted *R*-factor *wR* and goodness of fit *S* are based on *F*^2^, conventional *R*-factors *R* are based on *F*, with *F* set to zero for negative *F*^2^. The threshold expression of *F*^2^ > σ(*F*^2^) is used only for calculating *R*-factors(gt) *etc*. and is not relevant to the choice of reflections for refinement. *R*-factors based on *F*^2^ are statistically about twice as large as those based on *F*, and *R*-factors based on ALL data will be even larger.

### Fractional atomic coordinates and isotropic or equivalent isotropic displacement parameters (Å^2^)

**Table:**

	*x*	*y*	*z*	*U*_iso_*/*U*_eq_	
O1	0.3588 (2)	0.84256 (18)	0.76549 (10)	0.0943 (6)	
O2	0.2703 (2)	0.8468 (2)	0.86999 (14)	0.1240 (9)	
O3	0.69834 (12)	0.98409 (9)	0.67498 (6)	0.0495 (3)	
N1	0.42502 (13)	0.60777 (11)	0.72217 (7)	0.0421 (3)	
N2	0.58116 (12)	0.71200 (10)	0.68724 (7)	0.0381 (3)	
N3	0.3581 (2)	0.82279 (15)	0.82985 (10)	0.0720 (5)	
C1	0.2345 (2)	0.50148 (18)	0.63890 (11)	0.0643 (5)	
H1	0.2249	0.5090	0.6887	0.077*	
C2	0.1417 (3)	0.4507 (2)	0.59907 (14)	0.0823 (8)	
H2	0.0699	0.4249	0.6222	0.099*	
C3	0.1554 (3)	0.43820 (18)	0.52542 (13)	0.0763 (7)	
H3A	0.0929	0.4043	0.4988	0.092*	
C4	0.2609 (2)	0.47571 (15)	0.49166 (11)	0.0629 (5)	
H4	0.2704	0.4667	0.4420	0.075*	
C5	0.3536 (2)	0.52686 (14)	0.53059 (10)	0.0537 (4)	
H5	0.4251	0.5521	0.5068	0.064*	
C6	0.34175 (17)	0.54120 (12)	0.60510 (9)	0.0450 (4)	
C7	0.43445 (15)	0.60224 (12)	0.64747 (8)	0.0406 (3)	
C8	0.53212 (15)	0.66520 (12)	0.62466 (8)	0.0393 (3)	
C9	0.58588 (15)	0.67981 (14)	0.55083 (8)	0.0442 (4)	
C10	0.54220 (18)	0.75864 (16)	0.50552 (9)	0.0537 (4)	
H10	0.4809	0.8053	0.5219	0.064*	
C11	0.5900 (3)	0.7680 (2)	0.43568 (10)	0.0741 (7)	
H11	0.5611	0.8214	0.4058	0.089*	
C12	0.6789 (3)	0.6993 (3)	0.41067 (11)	0.0847 (8)	
H12	0.7111	0.7064	0.3641	0.102*	
C13	0.7207 (2)	0.6199 (2)	0.45434 (13)	0.0817 (8)	
H13	0.7803	0.5725	0.4370	0.098*	
C14	0.6749 (2)	0.60967 (18)	0.52423 (11)	0.0624 (5)	
H14	0.7039	0.5555	0.5535	0.075*	
C15	0.51231 (14)	0.67378 (12)	0.74329 (8)	0.0390 (3)	
C16	0.53902 (15)	0.70021 (13)	0.82028 (8)	0.0434 (3)	
C17	0.46705 (19)	0.76810 (14)	0.86142 (9)	0.0518 (4)	
C18	0.4959 (3)	0.78953 (19)	0.93326 (12)	0.0753 (7)	
H18	0.4461	0.8357	0.9597	0.090*	
C19	0.5983 (3)	0.7422 (2)	0.96475 (11)	0.0810 (8)	
H19	0.6188	0.7567	1.0126	0.097*	
C20	0.6698 (2)	0.6741 (2)	0.92610 (12)	0.0805 (7)	
H20	0.7392	0.6419	0.9477	0.097*	
C21	0.64027 (19)	0.6518 (2)	0.85425 (11)	0.0640 (5)	
H21	0.6892	0.6037	0.8288	0.077*	
C22	0.8477 (2)	0.58423 (16)	0.71153 (13)	0.0613 (5)	
H22	0.7936	0.5592	0.6758	0.074*	
C23	0.9023 (2)	0.51434 (18)	0.75950 (16)	0.0775 (7)	
H23	0.8833	0.4434	0.7564	0.093*	
C24	0.9841 (3)	0.5492 (2)	0.81139 (19)	0.0896 (8)	
H24	1.0202	0.5026	0.8440	0.108*	
C25	1.0122 (3)	0.6533 (3)	0.81481 (19)	0.0993 (9)	
H25	1.0702	0.6771	0.8489	0.119*	
C26	0.9552 (2)	0.7242 (2)	0.76789 (14)	0.0747 (6)	
H26	0.9737	0.7951	0.7719	0.090*	
C27	0.87162 (15)	0.69031 (15)	0.71554 (10)	0.0503 (4)	
C28	0.80645 (16)	0.76837 (14)	0.66683 (10)	0.0488 (4)	
H28A	0.7999	0.7390	0.6186	0.059*	
H28B	0.8581	0.8310	0.6637	0.059*	
C29	0.67331 (14)	0.79849 (12)	0.69335 (8)	0.0400 (3)	
H29	0.6811	0.8145	0.7450	0.048*	
C30	0.62316 (17)	0.89749 (13)	0.65685 (9)	0.0475 (4)	
H30A	0.6233	0.8880	0.6048	0.057*	
H30B	0.5365	0.9100	0.6721	0.057*	
H3	0.658 (2)	1.023 (2)	0.7087 (16)	0.078 (8)*	

### Atomic displacement parameters (Å^2^)

**Table:**

	*U*^11^	*U*^22^	*U*^33^	*U*^12^	*U*^13^	*U*^23^
O1	0.1086 (13)	0.1066 (14)	0.0678 (10)	0.0422 (12)	0.0073 (10)	0.0090 (10)
O2	0.1259 (17)	0.139 (2)	0.1075 (16)	0.0540 (16)	0.0522 (14)	0.0077 (15)
O3	0.0666 (7)	0.0394 (6)	0.0426 (6)	−0.0080 (5)	0.0105 (5)	−0.0074 (5)
N1	0.0531 (7)	0.0423 (7)	0.0309 (6)	−0.0089 (6)	−0.0013 (5)	0.0044 (5)
N2	0.0465 (6)	0.0352 (6)	0.0327 (6)	−0.0033 (5)	0.0020 (5)	0.0004 (5)
N3	0.0920 (12)	0.0620 (10)	0.0621 (11)	0.0134 (10)	0.0226 (9)	−0.0047 (9)
C1	0.0784 (12)	0.0682 (12)	0.0464 (9)	−0.0281 (11)	−0.0132 (9)	0.0115 (9)
C2	0.0859 (15)	0.0888 (17)	0.0722 (14)	−0.0435 (14)	−0.0203 (12)	0.0142 (13)
C3	0.0934 (16)	0.0622 (12)	0.0735 (14)	−0.0236 (12)	−0.0397 (13)	0.0008 (11)
C4	0.0942 (14)	0.0472 (10)	0.0472 (10)	−0.0038 (10)	−0.0249 (10)	−0.0031 (8)
C5	0.0724 (11)	0.0451 (9)	0.0437 (9)	−0.0043 (8)	−0.0091 (8)	−0.0001 (7)
C6	0.0598 (9)	0.0357 (7)	0.0394 (8)	−0.0048 (7)	−0.0119 (7)	0.0042 (6)
C7	0.0541 (8)	0.0361 (7)	0.0317 (7)	−0.0021 (7)	−0.0034 (6)	0.0030 (6)
C8	0.0513 (7)	0.0361 (7)	0.0307 (7)	−0.0014 (6)	−0.0013 (6)	−0.0003 (6)
C9	0.0524 (8)	0.0475 (8)	0.0328 (7)	−0.0097 (7)	0.0019 (6)	−0.0037 (6)
C10	0.0671 (10)	0.0579 (10)	0.0361 (8)	−0.0133 (8)	−0.0060 (7)	0.0053 (7)
C11	0.0982 (16)	0.0879 (16)	0.0363 (9)	−0.0388 (14)	−0.0085 (10)	0.0109 (10)
C12	0.0977 (16)	0.120 (2)	0.0368 (9)	−0.0439 (17)	0.0187 (11)	−0.0152 (12)
C13	0.0776 (13)	0.109 (2)	0.0584 (13)	−0.0133 (14)	0.0205 (11)	−0.0371 (14)
C14	0.0686 (11)	0.0683 (12)	0.0503 (10)	0.0008 (10)	0.0058 (8)	−0.0157 (9)
C15	0.0498 (7)	0.0369 (7)	0.0303 (7)	−0.0031 (6)	0.0016 (6)	0.0020 (6)
C16	0.0535 (8)	0.0449 (8)	0.0318 (7)	−0.0141 (7)	0.0004 (6)	0.0016 (6)
C17	0.0710 (10)	0.0452 (9)	0.0392 (8)	−0.0135 (8)	0.0101 (8)	−0.0021 (7)
C18	0.1157 (19)	0.0663 (13)	0.0440 (10)	−0.0335 (13)	0.0190 (11)	−0.0146 (9)
C19	0.1110 (18)	0.0966 (17)	0.0353 (9)	−0.0515 (16)	−0.0103 (11)	−0.0013 (10)
C20	0.0772 (13)	0.116 (2)	0.0484 (11)	−0.0226 (15)	−0.0201 (10)	0.0130 (12)
C21	0.0619 (10)	0.0845 (14)	0.0454 (10)	−0.0018 (10)	−0.0052 (8)	0.0060 (10)
C22	0.0624 (10)	0.0464 (10)	0.0753 (13)	0.0054 (8)	−0.0049 (9)	−0.0101 (9)
C23	0.0761 (13)	0.0500 (11)	0.1064 (19)	0.0169 (10)	−0.0039 (13)	−0.0032 (12)
C24	0.0769 (14)	0.0825 (17)	0.109 (2)	0.0233 (13)	−0.0197 (15)	0.0112 (16)
C25	0.0825 (16)	0.112 (2)	0.103 (2)	−0.0033 (16)	−0.0421 (16)	0.0016 (19)
C26	0.0761 (13)	0.0663 (13)	0.0817 (14)	−0.0173 (11)	−0.0171 (12)	−0.0039 (12)
C27	0.0423 (7)	0.0479 (9)	0.0606 (10)	−0.0009 (7)	0.0054 (7)	−0.0082 (8)
C28	0.0497 (8)	0.0442 (8)	0.0524 (9)	−0.0062 (7)	0.0083 (7)	−0.0017 (7)
C29	0.0486 (7)	0.0362 (7)	0.0351 (7)	−0.0058 (6)	0.0032 (6)	−0.0021 (6)
C30	0.0582 (9)	0.0385 (8)	0.0460 (8)	−0.0051 (7)	0.0000 (7)	0.0009 (7)

### Geometric parameters (Å, º)

**Table:**

O1—N3	1.216 (3)	C13—C14	1.385 (3)
O2—N3	1.226 (3)	C14—H14	0.9300
O3—C30	1.402 (2)	C15—C16	1.489 (2)
O3—H3	0.90 (3)	C16—C17	1.381 (3)
N1—C7	1.3858 (18)	C16—C21	1.385 (3)
N1—C15	1.308 (2)	C17—C18	1.389 (3)
N2—C8	1.4009 (19)	C18—H18	0.9300
N2—C15	1.3561 (19)	C18—C19	1.367 (4)
N2—C29	1.4762 (19)	C19—H19	0.9300
N3—C17	1.466 (3)	C19—C20	1.355 (4)
C1—H1	0.9300	C20—H20	0.9300
C1—C2	1.386 (3)	C20—C21	1.393 (3)
C1—C6	1.388 (3)	C21—H21	0.9300
C2—H2	0.9300	C22—H22	0.9300
C2—C3	1.378 (4)	C22—C23	1.384 (3)
C3—H3A	0.9300	C22—C27	1.381 (3)
C3—C4	1.363 (4)	C23—H23	0.9300
C4—H4	0.9300	C23—C24	1.364 (4)
C4—C5	1.379 (3)	C24—H24	0.9300
C5—H5	0.9300	C24—C25	1.364 (4)
C5—C6	1.395 (2)	C25—H25	0.9300
C6—C7	1.476 (2)	C25—C26	1.390 (4)
C7—C8	1.374 (2)	C26—H26	0.9300
C8—C9	1.489 (2)	C26—C27	1.379 (3)
C9—C10	1.389 (3)	C27—C28	1.509 (3)
C9—C14	1.388 (3)	C28—H28A	0.9700
C10—H10	0.9300	C28—H28B	0.9700
C10—C11	1.391 (3)	C28—C29	1.536 (2)
C11—H11	0.9300	C29—H29	0.9800
C11—C12	1.365 (4)	C29—C30	1.528 (2)
C12—H12	0.9300	C30—H30A	0.9700
C12—C13	1.369 (4)	C30—H30B	0.9700
C13—H13	0.9300		
			
C30—O3—H3	109.6 (17)	C17—C16—C21	117.09 (16)
C15—N1—C7	106.24 (13)	C21—C16—C15	118.52 (16)
C8—N2—C29	128.75 (12)	C16—C17—N3	120.76 (16)
C15—N2—C8	106.21 (12)	C16—C17—C18	122.0 (2)
C15—N2—C29	124.32 (12)	C18—C17—N3	117.27 (19)
O1—N3—O2	123.0 (2)	C17—C18—H18	120.2
O1—N3—C17	118.91 (17)	C19—C18—C17	119.5 (2)
O2—N3—C17	118.1 (2)	C19—C18—H18	120.2
C2—C1—H1	119.7	C18—C19—H19	120.0
C2—C1—C6	120.5 (2)	C20—C19—C18	119.94 (19)
C6—C1—H1	119.7	C20—C19—H19	120.0
C1—C2—H2	119.9	C19—C20—H20	119.7
C3—C2—C1	120.3 (2)	C19—C20—C21	120.6 (2)
C3—C2—H2	119.9	C21—C20—H20	119.7
C2—C3—H3A	120.1	C16—C21—C20	120.8 (2)
C4—C3—C2	119.8 (2)	C16—C21—H21	119.6
C4—C3—H3A	120.1	C20—C21—H21	119.6
C3—C4—H4	119.8	C23—C22—H22	119.2
C3—C4—C5	120.5 (2)	C27—C22—H22	119.2
C5—C4—H4	119.8	C27—C22—C23	121.5 (2)
C4—C5—H5	119.6	C22—C23—H23	119.9
C4—C5—C6	120.9 (2)	C24—C23—C22	120.2 (2)
C6—C5—H5	119.6	C24—C23—H23	119.9
C1—C6—C5	118.00 (17)	C23—C24—H24	120.4
C1—C6—C7	119.63 (16)	C25—C24—C23	119.2 (3)
C5—C6—C7	122.25 (17)	C25—C24—H24	120.4
N1—C7—C6	120.53 (14)	C24—C25—H25	119.6
C8—C7—N1	109.25 (13)	C24—C25—C26	120.8 (3)
C8—C7—C6	130.08 (14)	C26—C25—H25	119.6
N2—C8—C9	124.21 (14)	C25—C26—H26	119.7
C7—C8—N2	105.88 (12)	C27—C26—C25	120.6 (2)
C7—C8—C9	129.81 (14)	C27—C26—H26	119.7
C10—C9—C8	121.14 (16)	C22—C27—C28	122.24 (17)
C14—C9—C8	120.07 (16)	C26—C27—C22	117.5 (2)
C14—C9—C10	118.64 (17)	C26—C27—C28	120.17 (18)
C9—C10—H10	119.9	C27—C28—H28A	109.0
C9—C10—C11	120.1 (2)	C27—C28—H28B	109.0
C11—C10—H10	119.9	C27—C28—C29	113.05 (14)
C10—C11—H11	119.7	H28A—C28—H28B	107.8
C12—C11—C10	120.5 (2)	C29—C28—H28A	109.0
C12—C11—H11	119.7	C29—C28—H28B	109.0
C11—C12—H12	120.1	N2—C29—C28	112.95 (12)
C11—C12—C13	119.86 (19)	N2—C29—H29	106.6
C13—C12—H12	120.1	N2—C29—C30	110.98 (12)
C12—C13—H13	119.8	C28—C29—H29	106.6
C12—C13—C14	120.5 (2)	C30—C29—C28	112.52 (13)
C14—C13—H13	119.8	C30—C29—H29	106.6
C9—C14—H14	119.8	O3—C30—C29	110.62 (14)
C13—C14—C9	120.3 (2)	O3—C30—H30A	109.5
C13—C14—H14	119.8	O3—C30—H30B	109.5
N1—C15—N2	112.40 (13)	C29—C30—H30A	109.5
N1—C15—C16	124.36 (13)	C29—C30—H30B	109.5
N2—C15—C16	123.15 (13)	H30A—C30—H30B	108.1
C17—C16—C15	124.36 (16)		
			
O1—N3—C17—C16	−28.9 (3)	C10—C9—C14—C13	1.3 (3)
O1—N3—C17—C18	149.6 (2)	C10—C11—C12—C13	0.6 (4)
O2—N3—C17—C16	151.3 (2)	C11—C12—C13—C14	−1.0 (4)
O2—N3—C17—C18	−30.2 (3)	C12—C13—C14—C9	0.0 (4)
N1—C7—C8—N2	−1.39 (18)	C14—C9—C10—C11	−1.7 (3)
N1—C7—C8—C9	174.96 (16)	C15—N1—C7—C6	−174.64 (14)
N1—C15—C16—C17	−78.5 (2)	C15—N1—C7—C8	1.39 (18)
N1—C15—C16—C21	99.5 (2)	C15—N2—C8—C7	0.87 (16)
N2—C8—C9—C10	−91.6 (2)	C15—N2—C8—C9	−175.75 (15)
N2—C8—C9—C14	92.9 (2)	C15—N2—C29—C28	123.81 (16)
N2—C15—C16—C17	105.10 (19)	C15—N2—C29—C30	−108.76 (16)
N2—C15—C16—C21	−76.9 (2)	C15—C16—C17—N3	−2.0 (2)
N2—C29—C30—O3	168.26 (12)	C15—C16—C17—C18	179.64 (17)
N3—C17—C18—C19	−178.64 (19)	C15—C16—C21—C20	179.7 (2)
C1—C2—C3—C4	0.2 (4)	C16—C17—C18—C19	−0.2 (3)
C1—C6—C7—N1	9.2 (3)	C17—C16—C21—C20	−2.2 (3)
C1—C6—C7—C8	−165.92 (19)	C17—C18—C19—C20	−0.7 (3)
C2—C1—C6—C5	−1.0 (3)	C18—C19—C20—C21	0.1 (4)
C2—C1—C6—C7	175.2 (2)	C19—C20—C21—C16	1.4 (4)
C2—C3—C4—C5	−0.5 (4)	C21—C16—C17—N3	−179.99 (17)
C3—C4—C5—C6	0.1 (3)	C21—C16—C17—C18	1.6 (3)
C4—C5—C6—C1	0.7 (3)	C22—C23—C24—C25	−0.7 (5)
C4—C5—C6—C7	−175.39 (17)	C22—C27—C28—C29	81.6 (2)
C5—C6—C7—N1	−174.82 (16)	C23—C22—C27—C26	1.9 (3)
C5—C6—C7—C8	10.1 (3)	C23—C22—C27—C28	−176.0 (2)
C6—C1—C2—C3	0.6 (4)	C23—C24—C25—C26	2.3 (5)
C6—C7—C8—N2	174.14 (16)	C24—C25—C26—C27	−1.8 (5)
C6—C7—C8—C9	−9.5 (3)	C25—C26—C27—C22	−0.3 (4)
C7—N1—C15—N2	−0.83 (18)	C25—C26—C27—C28	177.6 (2)
C7—N1—C15—C16	−177.60 (16)	C26—C27—C28—C29	−96.2 (2)
C7—C8—C9—C10	92.6 (2)	C27—C22—C23—C24	−1.4 (4)
C7—C8—C9—C14	−82.8 (2)	C27—C28—C29—N2	−69.13 (18)
C8—N2—C15—N1	−0.02 (18)	C27—C28—C29—C30	164.25 (14)
C8—N2—C15—C16	176.79 (15)	C28—C29—C30—O3	−64.08 (18)
C8—N2—C29—C28	−67.3 (2)	C29—N2—C8—C7	−169.57 (14)
C8—N2—C29—C30	60.1 (2)	C29—N2—C8—C9	13.8 (2)
C8—C9—C10—C11	−177.23 (16)	C29—N2—C15—N1	170.96 (14)
C8—C9—C14—C13	176.89 (19)	C29—N2—C15—C16	−12.2 (2)
C9—C10—C11—C12	0.8 (3)		

### Hydrogen-bond geometry (Å, º)

Cg1 and Cg2 are the centroids of the C9–C14 and C22–C27 rings, respectively.

**Table:**

*D*—H···*A*	*D*—H	H···*A*	*D*···*A*	*D*—H···*A*
C5—H5···*Cg*1	0.93	2.84	3.715 (2)	157
C21—H21···*Cg*2	0.93	2.81	3.501 (2)	132
O3—H3···N1^i^	0.90 (3)	1.89 (3)	2.7935 (17)	179 (3)

Symmetry code: (i) −*x*+1, *y*+1/2, −*z*+3/2.
